# Preparation, Characterization, and Antibacterial Properties of Cu-Fibreboards

**DOI:** 10.3390/ma16216936

**Published:** 2023-10-28

**Authors:** Lyubomir Aleksandrov, Nadezhda Rangelova, Nevena Lazarova-Zdravkova, Nelly Georgieva, Mirela Dragnevska, Sanchi Nenkova

**Affiliations:** 1 Institute of General and Inorganic Chemistry, Bulgarian Academy of Sciences, 1113 Sofia, Bulgaria; 2 Department of Industrial Safety, University of Chemical Technology and Metallurgy, 1756 Sofia, Bulgaria; rangelovang@gmail.com; 3 Department of Biotechnology, University of Chemical Technology and Metallurgy, 1756 Sofia, Bulgaria; n.a.lazarova@gmail.com (N.L.-Z.); neli@uctm.edu (N.G.); 4 Department of Pulp, Paper and Printing Arts, University of Chemical Technology and Metallurgy, 1756 Sofia, Bulgaria; mireladr@mail.bg (M.D.); nenkova@uctm.edu (S.N.)

**Keywords:** fibreboards, modification, copper nanoparticles, antibacterial activity

## Abstract

In the present study, copper modified fibreboards were prepared and their existing phase, morphology, and antibacterial behaviour were investigated. The copper content and the physical and mechanical properties of fibreboards (thickness, bending strength, and swelling) were determined. X-ray diffraction analysis (XRD) showing diffraction peaks typical for cellulose, Cu_2_S, and Na_2_SO_4_, depended on the preparation conditions. The average size of the Cu_2_S crystals varied between 20 and 50 nm. The morphology of the obtained fibreboards, as well as the size and shape of copper particles, were observed by scanning electron microscopy (SEM) and transition electron microscopy (TEM). The antibacterial activity was tested against Gram-positive (*Bacillus subtilis* 3562) and Gram-negative (*Escherichia coli* K12 407) bacteria. The tests showed that the materials had higher antibacterial activity against *E. coli*, which depended on their preparation conditions. Based on these results, the obtained copper fibreboards can be used as antibacterial agents in the packaging and building industry.

## 1. Introduction

Fibreboards are structural and decorative materials, fibrous homogeneous panels made from lignocellulosic materials that are combined most commonly with a synthetic resin and then bonded together under heat and pressure [1,2,3]. It is believed that, of all wood materials, the panel of fibreboards has the uniform structure because they are made of very fine and flexible particles, which determine the specific physical and mechanical parameters [4]. The manufactured fibreboards can find applications in various industries, such as the furniture industry, the automobile industry, the building industry, packaging, and electronic device applications [5,6,7]. An essential part of the woodworking sector is the production of wood composites, including wood fibreboards. The consumption of such composite materials in 2022 reached about 14.71 million m3, mainly determined in different industries [8]. The main component of the fibreboards is fibrous materials; as with lignocellulose materials, many sources can be used: corn biomass, wood fibres, lignin, and others [1,2,3,9,10]. Lignosulfonates are successfully used as binders used in the production of fibreboards. The new tendency is the manufacturing of fibreboards, where the waste technical hydrolysis lignin is used as an additive to the wood mass and its physical and mechanical properties are investigated [2,11,12]. In order to improve the thermal, physical, and mechanical properties of the fibreboards, various additives are often used (CuO, ZnO, Al_2_O_3_, nanoparticles, multi carbon nanotubes, and others). The improved properties are the result of the final composite becoming stronger due to the covering of unwanted cracks and voids by the used additive [10,13,14,15,16,17]. For instance, Alabduljabbar et al. [10] reported that when the concentration of alumina nanoparticles increased, the mechanical properties of the panels were significantly positively affected.

Due to contact with water, fibreboards used in humid environments have low durability. For increased durability and antimicrobial attack reduction, materials with metallic nanoparticles (such as ZnO) were developed [13]. Kandelbauer and Widsten described [18] the basic strategies to achieve antimicrobial properties on the surface of materials. As protection coatings against the growth of microorganisms, antimicrobial additives of organic and inorganic origin were used [18]. Inorganic additives include metals such as silver, copper, and zinc in different forms (salt; nano-sized), TiO2, or others. It was reported that the addition of nanoparticles to the surface of materials leads to improved antimicrobial resistance [18,19]. In order to form particles and avoid clustering, it is necessary to stabilize them in a suitable matrix, which will ensure a homogeneous structure and their uniform distribution [20,21,22,23,24,25]. It was reported that copper nanoparticles are a new generation of wood preservative materials that work against decay fungi when they are used instead of conventional copper [17]. Many authors reported the antimicrobial effect of copper-based composite coatings against Gram-positive and Gram-negative bacteria [26,27]; others focused on films containing copper nanoparticles (CuNPs) [28]. At the same time there is scarce of information about the antibacterial effect of nanoparticles with the Cu_2_S active form. Recently, the antibacterial activity of CuS/Cu_2_S under near-infrared (NIR) irradiation was reported [29]. In our previous studies, the mechanism of the incorporation of Cu_2_S into wood fibres by the coordinative bonding of copper ions with lignocelluloses materials and technology for wood fibre plates was reported [1,30]. The good prerequisite for the successful incorporation of copper particles can be explained by the fact that lignocellulosic materials contain a large number of functional groups from the building blocks of wood [30]. Considering the availability of few scientific works related to the antibacterial activity of wood-modified fibreboards, as well as our previous experience with the antimicrobial efficiency of various composite materials, motivated us to check the ability to modify fibreboards with copper and to clarify their antibacterial activity at different preparation conditions.

## 2. Materials and Methods

### 2.1. Material and Fibreboards Preparation

Five types of fibreboards were obtained using as start materials wood fibres (WF, Welde Bulgaria AD, Troyan, Bulgaria) and a two-component cupri reduction system as described previously [1,27]. CuSO_4_*5H_2_O (Merck KGaA, Amsterdam, The Netherlands) was used as the metal precursor to produce CuNps. On the other hand, Na_2_S_2_O_3_*5H_2_O (Valerus Ltd., Sofia, Bulgaria) was used as a reductor. 

The main stages of obtaining of the fibreboards are summarized as follows:

The supplied wood fibres were diluted to a concentration of 4–6% with water. The next step was gluing, where 15% phenol formaldehyde resin as a binding agent (46.0–47.0% dry solids content, viscosity 300–450 mPa.s, Dynea, Bucharest, Romania) and 10% paraffin suspension as a water-repelling agent (Lukiol, Burgas, Bulgaria) in 1% of absolute dry fibres were used. After that, the wood fibre mass was further diluted by water to 1.3–1.8%. Using a dewatering process, the wood carpet was obtained. The moulding had a size of 30 × 30 cm, and the water solutions of the modifying mixture were applied to the surface of the obtained wood carpet [1]. The modification process was performed at 45% of the mixture to the absolute dry wood fibres and corresponding to the molar ratio between the components (CuSO_4_*5H_2_O:Na_2_S_2_O_3_*5H_2_O): 1:1, 1:1.5, and 1:2. The duration of residence of the applied modifying mixture on the carpet before the pressing process was 7, 12, and 17 min, respectively. The resulting modified wood carpets were pressed in a laboratory press at 170 °C for 10 min at high pressure (~4 MPa). The density of the obtained fibreboards was not less than 900 kg/m^3^ [6].

### 2.2. Physical and Mechanical Characterizations of the Fibreboards

Physical and mechanical properties such as bending strength, thickness, and swelling were determined in accordance with national standards: BDS EN 310:1999, BDS EN 317:1998, and BDS EN 324-1:2001 [31,32,33]. The copper content in the samples was determined by inductively coupled plasma atomic emission spectroscopy ((ICP)—Prodigy High Dispersion ICP Spectrometer—Teledyne Leeman Labs, Mason, OH, USA), by thermal and acid decomposition methods. The samples were first immersed in concentrated HNO_3_ solutions, ensued to dissolve all metal nanoparticles in the solutions. The resultant solutions were then sampled and quantified on ICP. All tests were performed three times and the average values are reported.

### 2.3. Structural and Morphological Characterizations of the Fibreboards

X-ray diffraction patterns were used to investigate the crystalline phases that appear in the materials. A Bruker D8 Advance diffractometer, Karlsruhe, Germany, was used at Cu Kα radiation in the 10 < 2θ < 80 range. The diffraction peaks of the samples were assigned using HighScore XRD analysis software version 3.0.4. The Debye Scherrer equation, D = Kλ/βcosθ, was used to calculate the crystalline size of the crystals phase, where D is the crystalline size, K represents the Scherrer constant (0.98), λ is the X-ray wavelength (1.5418), and β is the full width at half maximum. The thermal stability of the obtained fibreboards was examined by parallel differential thermal analysis and thermogravimetry (DTA/TG) with the Seteram Labsysis Evo 1600 instrument, Lyon, France, in the temperature range of 25–650 °C at the heating rate of 10 K/min in the air atmosphere. Optical microscopy images were recorded using a Light Microscope BOEKO, Boeckel & Co. GmbH & Co. KG, Hamburg, Germany, at magnification of 5×. SEM images were recorded using the microscope JEOL JSM 6390, Oxford instrument, JEOL Ltd., Tokyo, Japan, at an accelerating voltage of 20 kV. Prior to analysis, the samples were coated with gold using a JEOL JFC-1200 coater in order to improve the topographic examination of the samples. The energy dispersive X-ray spectroscopy (EDX) images were obtained using the INCA Oxford instrument. TEM images were recorded using the transmission electron micrographs using a JEOL-2100, Oxford instrument, JEOL Ltd., Tokyo, Japan, at an accelerating voltage of 200 kV. The specimen was prepared by grinding and dispersing the powder in ethanol by ultrasonic treatment for 6 min. The suspension was dripped on a standard carbon/Cu grid.

### 2.4. Antibacterial Activity of the Fibreboards

The model bacterial strains used in this study included the Gram-positive bacteria *Bacillus subtilis* NBIMCC 3562 and the facultative anaerobic Gram-negative *Escherichia coli* K12 NBIMCC 407. The strains were obtained from the Bulgarian National Bank of Industrial Microorganisms and Cell Cultures. The cultures were grown, subcultured, and maintained in Luria–Bertani (LB) medium and stored at 4 °C. For the experiment, a single colony of each organism was inoculated into 50 mL of LB broth and incubated overnight (24 h) at 30 °C for *B. subtilis* 3562 and 37 °C for *E. coli* K 12 with shaking at 200 rpm. Then, 10 mg from each tested material were placed in a flask with the investigated strains. The control contained only a bacterial culture.

Bacterial growth in the presence of the tested materials was monitored by optical density measurements at 610 nm (OD_610_) over 24 h at hourly intervals using a UV-Vis spectrophotometer (VWR UV-1600, VWR Corporate Headquarters, Radnor, PA, USA). The growth curves of the test organisms were analyzed graphically as a plot of OD_610_ versus contact time. The bacterial growth-inhibiting effect of the obtained materials was further confirmed by plating on LB agar plates, and the assay was based on the reduction in viable cells after exposure to the materials. Following 24 h of strain cultivation in the presence of the test materials, 100 µL of bacterial broth suspension was seeded on LB agar plates. Samples containing only bacteria were used as controls. The plates were incubated for 24 h at 30 °C for *B. subtilis* and 37 °C for *E. coli* K12, followed by counting the number of colonies on the plate. The measurement of the antibacterial activity of fibreboards was calculated according to Rangelova et al. [21]:

$$Cell\;reduction=\left(1-\frac{Test\;sample\left(CFU/mL\right)}{Control\left(CFU/mL\right)}\right)\times 100\%.$$

The model strains were cultivated for 24 h with the fibreboards, and the release of copper ions in the suspensions was determined with the help of ICP analysis. All determinations were performed three times in duplicate sets and the average values are reported.

## 3. Results and Discussion 

Modified fibreboards containing copper ions were developed using the two-component reduction system CuSO_4_*5H_2_O:Na_2_S_2_O_3_*5H_2_O at the different ratios mentioned in Table 1. Visually, the colour on the surface of the obtained fibreboards changed from brown for the samples without copper to black depending on the preparation condition of the samples. The fibreboard preparation conditions and average results for copper content, physical, and some mechanical properties are given in Table 1. As can be seen, the molar ratio of the modifying mixture (CuSO_4_*5H_2_O:Na_2_S_2_O_3_*5H_2_O) has an effect on the copper content of the samples. Fibreboards F3 and F4 were characterized with lowest copper content and were obtained at the same molar ratio (1:2). This means that the duration of residence of the applied modifying mixture on the carpet before pressing did not significantly influence the copper content in the samples. The most suitable conditions for the highest copper content were obtained by applying the conditions for preparation of F1 fibreboard (see Table 1). 

In Table 1, some physical and mechanical properties of the obtained fibreboards can also be found. If it is compared, there are no significant differences in the values of the thickness (mm) between the samples. The other two parameters (bending strength and swelling) decreased compared to the unmodified fibreboards, but nevertheless, the values were within the accepted range of requirements according to European and national standards [34,35]. Similar results for the reduction in some physical properties for fibreboards modified by other metal nanoparticles (zinc and silver) were reported [36]. It was noticeable that sample F1 has the lowest value of bending strength and swelling due to the stable interaction between the matrix and the modifier agent. We consider that with the increase in copper content, free functional groups of lignocellulose materials are occupied with copper ions, and in this case, it is more difficult for phenol formaldehyde resin to affect a better connection with WF.

**Figure 1:**
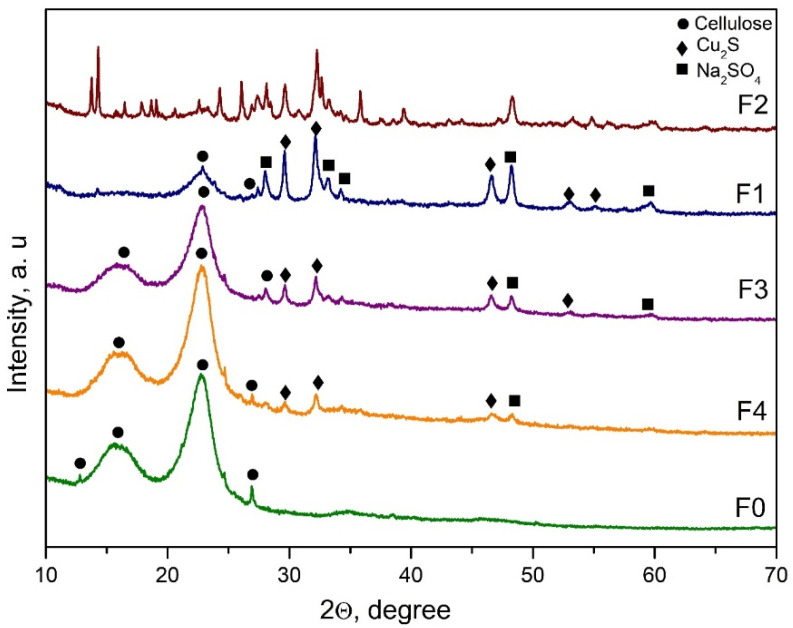
XRD patterns of unmodified and copper modified fibreboards.

XRD patterns on the surface of the investigated fibreboards are presented in Figure 1. As can be seen, typical sharp peaks around 2θ = 14, 16, and 22, which are characteristic for cellulose, appeared for all samples except for sample F2 [37,38,39]. On the other hand, the modified fibreboards containing copper showed some diffraction lines that correspond to the following crystal phases: Cu_2_S (ICSD 98-062-8818) and Na_2_SO_4_ (ICSD 98-010-0458). The obtained phases proved the proposed probable mechanism of the reduction process that was reported previously by us [30]. For the samples, the F3 and F4 diffraction peaks characteristic of the cellulose were dominating compared to the other characteristic diffraction lines. For the F2 fibreboard, it was difficult to identify the cellulose diffraction peaks, but several crystal phases appeared. Clearly, sample F1 was characterized by a predominating Cu_2_S crystal phase compared to the other fibreboards. Obviously, for the copper content, the ratio of the modified mixture was a critical parameter. On the other hand, for successful preparation of Cu_2_S, both parameters, the ratio of the modified mixture and the duration of residence of the applied modifying mixture on the carpet before pressing, were important. In addition, the crystal size of the formed Cu_2_S was calculated by the Debye Scherrer equation. The average size of the crystals from copper sulfide varied between 20 and 50 nm. The smallest particles were calculated in sample F4, around 20 nm. For sample F1, the average size of Cu_2_S was 31 nm, while in samples F2 and F3, the particles had sizes at around 35 and 50 nm, respectively. 

**Figure 2:**
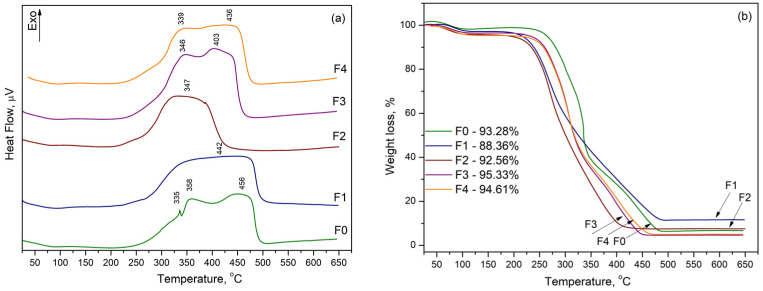
DTA curves (**a**) and TG curves (**b**) of unmodified and copper modified fibreboards.

In order to determine the thermal stability of modified fibreboards the DTA/TG analysis was performed (Figure 2). The small endo effect characteristic for all DTA curves (Figure 2a) at ~70 °C was due to the moisture and volatile matters evaporation. This effect corresponded to the initial reduction in weight in the samples that appears in TG curves (Figure 2b) and the mass loss varied from 2 to 5 wt.% depending on the copper content. The next broad and intense exothermic effects may correspond to the simultaneous degradation of the lignocellulose components [40,41,42,43]. Cellulose degradation occurred in the temperature range between 200 °C and 400 °C, while lignin degradation was achieved at higher temperatures [36,37,38]. All samples showed quite similar thermogravimetry curves and thermal degradation behaviour. The mass loss varied from 88 to 95 wt.%.

**Figure 3:**
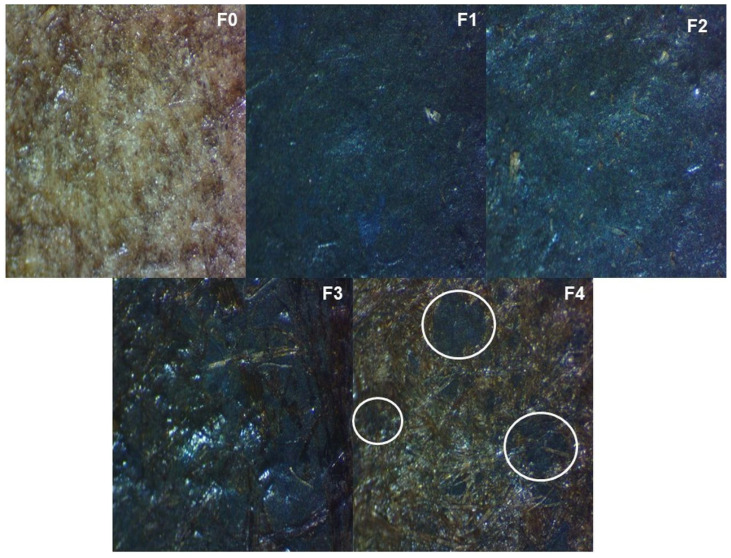
Optical microscopy images on the surface of unmodified and copper modified fibreboards at magnification 5×.

The microscope reflection images of the surface of the obtained fibreboards are presented in Figure 3. The unmodified sample (F0) was characterized by a typical fibrous structure. A non-homogeneous distribution of copper on the sample surface for the F4 fibreboard was observed (see white rings for sample F4). To a lesser extent, this problem is also seen in sample F3. A densification of the surface and a more even distribution of the layer without the presence of aggregates for F1 and F2 fibreboards were observed. According to the presented images, the F1 fibreboard was characterized by the most homogeneous, flat, and dense surface. Obviously, the ratio between the modified mixture played an important role for homogenous distribution of copper on the samples surface. 

**Figure 4:**
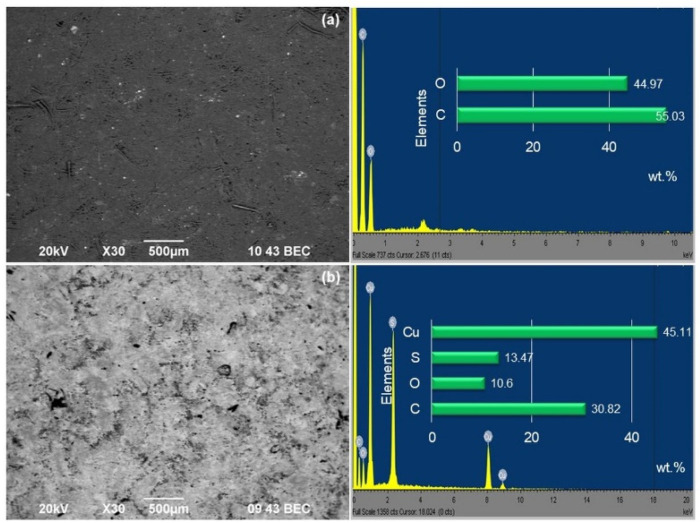
SEM and EDX images of unmodified—F0 (**a**) and copper-modified—F1 (**b**) fibreboards.

The morphology of the fibreboards and the size and shape of the copper crystallites were observed by SEM and TEM analyses. Due to some limitations, we decided to compare the unmodified fibreboard (F0) and the sample with the best homogeneity (F1). The SEM micrographs and EDX analysis are presented in Figure 4. The observed surfaces look like a pressed “carpet” with a fibrous structure. The difference between the investigated samples was in the presence of homogenously distributed glowing objects, which corresponded to the copper particles. It is also seen that copper nanoparticles were able to cover unwanted cracks and voids very well, as mentioned in the literature [10,13,14,15,16,17]. The provided elemental analysis showed the presence of carbon and oxygen on the surface of the F0 fibreboard while for sample F1, the presence of copper and sulphur was also found.

**Figure 5:**
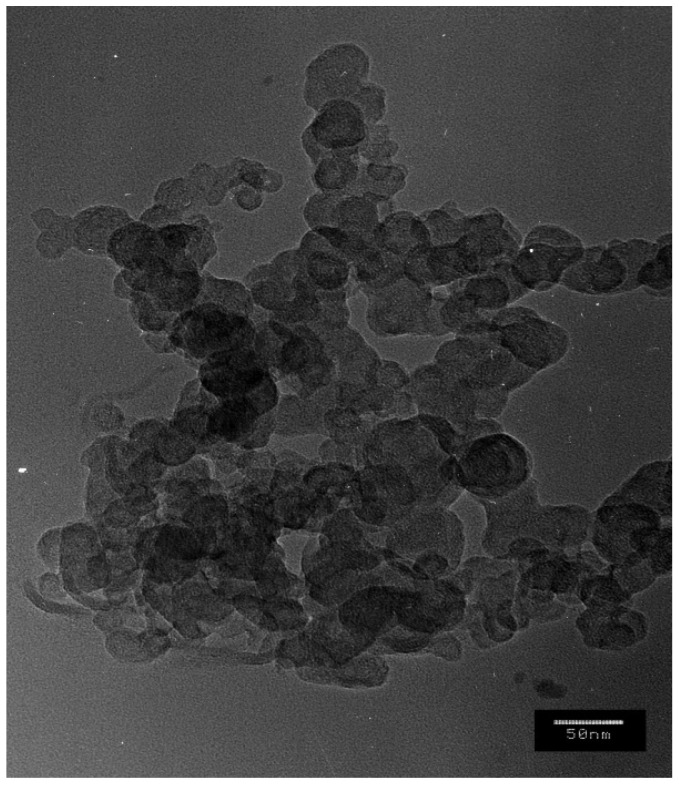
TEM image of copper-modified fibreboard F1 (bar is 50 nm).

In addition to SEM analysis, in order to determine the size and shape of copper particles, TEM analysis was performed (Figure 5). The TEM image of powdered flakes from the surface of the F1 fibreboard showed spherical-shaped nanoparticles with an average size of 35 nm. As can be seen, the majority of nanoparticles have the same shape and the same size. The obtained results are in good agreement with the results of the X-ray analysis. Indeed, the fibreboard used and the modification methodology used were suitable for the formation and deposition of copper nanoparticles.

The antibacterial activity of the obtained fibreboards was investigated against the model strains of the Gram-positive (*B. subtilis*) and Gram-negative (*E. coli* K12) bacteria. For this purpose, two methods were used: one based on building growth curves and the other on tracking the number of the colony-forming unit (CFU) of microorganisms after treatment with the analyzed materials.

**Figure 6:**
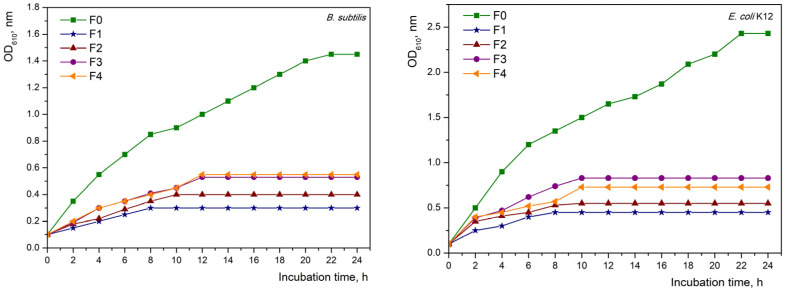
Bacterial growth curves of *B. subtilis* and *E. coli* K12 in the presence of fibreboards.

The first method for determining the antimicrobial properties of the obtained materials was by tracking the growth of microorganisms in a batch culture and constructing growth curves. In Figure 6 are presented the batch growth curves of *E. coli* and *B. subtilis* in the presence of the fibreboards for 24 h. The growth of both strains in the presence of fibreboard F0 was in the typical growth phases for microorganisms; the lag and exponential phases were clearly expressed. The presence of fibreboards of different copper content (from 1 to 4) changed the form of the growth phases, in which a significant lengthening of the lag phase and shortening of the exponential phase were observed in both studied strains. The most notable change in the growth phases was observed in fibreboard F1, where individual phases were difficult to distinguish.

It is clear from the growth curves for both strains that the presence of the resultant boards (in all variants) had a greater impact on *E. coli* growth than *B. subtilis* growth. This is most likely caused by the varied accessibility of copper ions in the fibreboards as well as the different cell wall composition of Gram-positive and Gram-negative bacteria. The obtained results suggest that *E. coli* is more sensitive than *B. subtilis*. It was noted that the existence of copper nanoparticles has an impact on the development of microorganisms, as well as the contact time between them. The increased amount of the metal ions in the materials caused the growth phases to last longer and the growth to be delayed, which was proof of their inhibitory impact.

**Figure 7:**
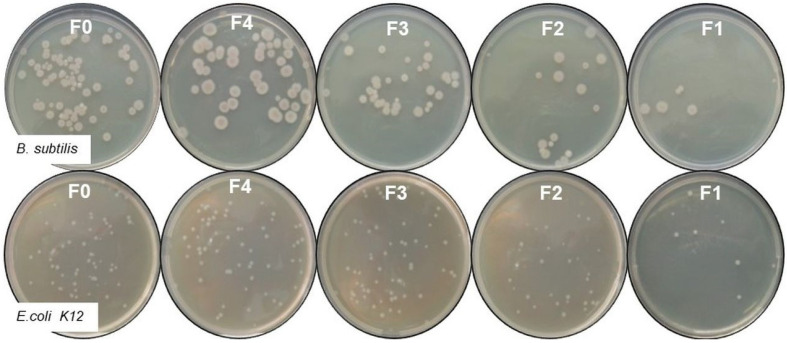
Antibacterial activity of tested fibreboards against model bacteria.

Another method of monitoring the antimicrobial activity of wood fibreboards was by monitoring the CFU counts (Figure 7). Petri dishes with single colonies of *E. coli* and *B. subtilis*, respectively, grown after exposure to boards with different copper contents are presented. The results show that the most cells grew in the control variant (F0) and fibreboard F4, which had the lowest copper content. In the remaining photos (fibreboards 1 to 3), a directly proportional decrease in the number of cells with increasing copper concentration was observed. These results correlate with the results from the growth curves, and it can be seen that E. coli exhibited a much higher sensitivity to the materials compared to *B. subtilis.* The highest inhibition was observed for fibreboard F1, where for *E. coli*, it was 84.6%, and for *B. subtilis*, it was 70.5%, respectively. In Table 2 are presented the results for all of the samples, together with the concentration of the copper nanoparticles in the medium. The similar results for copper nanoparticles highly sensitive to *E. coli* compared to the other microorganism were reported by Ramyadevi and co-workers [44].

The mechanism of the biocidal action of CuNps may be explained by the fact that CuNps release Cu(II) ions upon contact with moisture. These copper ions could bind with the -COOH and -SH groups of protein molecules of the bacterial cell wall. M. Crace et al., 2009 [45] obtained similar results by investigating copper alginate-cotton cellulose fibres. The surface of bacterial cells is negatively charged due to excess amounts of carboxyl groups in lipoproteins. At the same time, copper ions obtained from the nanoparticles in the liquid nutrient medium are positively charged. Therefore, adhesion and bioactivity are assumed to occur due to electrostatic forces. Peptidoglycans are negatively charged molecules that also bind copper ions released in the liquid growth medium. As a Gram-negative bacterium, *E. coli* may allow a greater amount of Cu^2+^ to reach the plasma membrane, but in most cases, it is less susceptible to antibacterial agents and antibiotics than Gram-positive bacteria [46].

The highest inhibition was observed for fibreboard F1, where for *E. coli* it was 84.6% and for *B. subtilis* it was 70.5%, respectively. In Table 2 are presented the results for all of the samples, together with the concentration of the cooper nanoparticles in the medium.

As it is known, copper is an essential biocide. It is important to mention that copper alone fails to protect wood against fungi. For copper to be active, a second metal, such as chromium or arsenic, needs to be present in the system [47]. It was reported that the presence of copper nanoparticles instead of conventional copper improved the durability of wood against decay fungi, and it is not necessary for a second metal to be present in the system [17,47]. Spherical nanoparticles were reported to have a well-developed surface that results in good antibacterial properties [48,49]. Very recently, the excellent antibacterial activity of nano-derived CuS/Cu_2_S against E. coli under near-IR light was reported [29]. The authors explained this excellent antibacterial activity with the presence of efficient interfacial charge separation and the generation of much more reactive oxygen species (ROS) [29]. Obviously, for efficient antibacterial ability, the copper content, the shape, and nano dimension were the key parameters. The presence of homogenously distributed nano Cu_2_S was also important since this crystalline phase was characterized by excellent antibacterial properties [29]. The results show that the non-stoichiometric ratio of the modifying agents (CuSO_4_*5H_2_O:Na_2_S_2_O_3_*5H_2_O) led to the presence of several copper-containing crystalline phases that were not identified by us. This means that the stoichiometric amounts of the modifying mixture also affect the successful synthesis of Cu_2_S. Apparently, F1 fibreboard possessed the best nano-crystallinity regarding Cu_2_S crystal phases (Figure 1), as well as the best stoichiometric ratio. On the other hand, this sample had enhanced thermal stability and a flat, homogeneous distribution of Cu_2_S, and a denser surface compared to the other investigated specimens. Moreover, the TEM analysis (Figure 5) of fibreboard F1 showed the presence of spherical copper nanoparticles, with an average size of 35 nm (31 nm calculated by Debye Scherrer equation), formed by self-organizational processes. It can be summarized that the degree of inhibition of the tested microorganisms was directly dependent on the concentration of copper nano-particles presented in the medium as well as the contact time between them. Considering our results, the efficient antibacterial behaviour of the F1 fibreboard was obtained even without additional light exposure [29].

The results show that the obtained materials are suitable for manufacturing of packaging and furniture. Due to contact with water, fibreboards used in humid environments have low durability against fungal attacks. This fact gives us the idea to investigate the antifungal activity of the obtained fibreboards in the future.

## 4. Conclusions

The copper-modified fibreboards were prepared and their morphology, thermal stability, and antibacterial behaviour were investigated. The modification process was carried out by varying the copper reduction system. The copper content was determined and the physical and mechanical properties of fibreboards (thickness, bending strength, and swelling) were also presented. It was found that as the copper content increased, the bending strength and swelling decreased. Nevertheless, all mechanical parameters were in accordance with the European and national standards. Spherical nanoparticles, with an average size of 35 nm, were observed on the surface of the F1 fibreboard. The antibacterial activity was tested against model Gram-positive and Gram-negative bacteria. The results indicate that the antimicrobial activity of the materials depended on the copper content and preparation conditions. On the other hand, the copper content depended on the ratio of the modified mixture applied to the surface of the obtained wood carpet. In this regard, the fibreboards F1 showed the best antibacterial activity. The tests indicated that the materials had pronounced antibacterial performance against *E. coli.*

## Figures and Tables

**Table 1:** Fibreboard production conditions (copper reduction system; duration of residence of the applied modifying mixture on the carpet before pressing) and characteristics.

Fibreboards	Conditions 45% Reagents to WF and 170 °C	Copper Content in Samples, %	Physical and Mechanical Properties		
			Thickness, mm	Bending Strength, MPa	Swelling, %
**F0**	-	0.01	2.8	49.78	34.48
**F1**	ratio 1:1; 17 min	1.07	3.0	33.30	19.35
**F2**	ratio 1:1.5; 12 min	0.76	2.9	41.62	25.92
**F3**	ratio 1:2; 7 min	0.36	2.8	44.01	32
**F4**	ratio 1:2; 17 min	0.40	2.9	40.84	34.61

**Table 2:** Antibacterial test results and copper release after cultivation for 24 h.

Sample	*B. subtilis*			*E. coli*		
	CFU/mL	Inhibition, %	Copper Release, mg/L	CFU/ mL	Inhibition, %	Copper Release, mg/L
F0	68 × 10^8^	0	0.044	2.6 × 10^10^	0	0.027
F1	20.1 × 10^8^	70.5	12.67	0.4 × 10^10^	84.6	11.9
F2	35 × 10^8^	48.5	8.909	0.6 × 10^10^	77	8.093
F3	47.2 × 10^8^	31	2.050	1.6 × 10^10^	38.5	1.769
F4	39.2 × 10^8^	42.4	2.80	1.6 × 10^10^	38.5	3.797

## Data Availability

Not applicable.
